# Supplementary material for: The Relevance of Telomerase and Telomere-Associated Proteins in B-Acute Lymphoblastic Leukemia
Source: Genes (Basel). 2023 Mar 10;14(3):691. doi: 10.3390/genes14030691 (PMC10048576; doi:10.3390/genes14030691)
Supplement: Supplementary file 1 [file genes-14-00691-s001.zip › genes-2244162-supplementary.pdf]

Table S1: Markers associated to canonical and non-canonical functions of telomerase

GENE	CELL TESTED	RELATION TO TERT	REFERENCE
ADP RIBOSE	MCF-7	Protect against DNA damage	[84]
AP1	U2OS	Downregulates *hTERT* expression	[85]
B CATENIN	AGS, MCF7, 293T, MCF10A, HCT116, and LS174T	Increase the expression of TERT mRNA and telomerase activity (TA)	[86]
BAP1	21NT	Associated with a significant reduction in *hTERT* expression levels	[87]
B-ARRESTIN	Bone Marrow	Participate in regulation of *hTERT* transcription and are related with senescence	[88]
BCL-2	CTLL-2	Increased telomerase activity	[89]
BCL-X1	HEK 293T/17, U2OS, and HeLa	Mitochondrial pathway of apoptosis	[90]
BECN1	U87	Downregulation of *hTERT* results in autophagy by suppressing *BECN1/beclin-1* promoting cell death	[91]
BPTF	Hep3B, HepG2, SNU-449 and L-O2	Promote cellular growth	[92]
BRG1	Xenopus laevis embryos	Transcriptional regulation and chromatin remodeler	[93]
CBX4	breast cancer cells	Regulation of gene expression promoting migration and invasion	[94]
CDC5L	CRC cells	*hTERT* promoter-binding protein and *CDC5L* knockdown inhibited tumor growth by reduce *hTERT* expression	[95]
CDK1	HEK-293T	Contribute to cancer progression in a telomere independent manner.	[96]
CDX2	Gastric intestinal metaplasia	*hTERT* works activating *NF-kB* pathway and upregulating *CDX2*	[97]
C-MYC	EREB	Upregulation of the *hTERT* expression and TA	[98]
CTCF	A427, A549 and H838	Repress hTERT expression.	[99]
DTX2	HEK293T, HeLa, HT-1080, DLD1, Hs578T and MDA-MB-231	*DTX2* promote *hTERT* transcription.	[100]
E2F	SCC25	Act as repressor and activator of *hTERT* expression	[101]
E2F1	fibroblast cells	Overexpression of E2F1 downregulated *hTERT* activity.	[102]
EGF	A549, H129, and NSCLC	Upregulated *RFPL3* and *hTERT* protein expression rising expression of hTERT	[103]
ENDOG	Fresh peripheral blood	Participate in alternative mRNA splicing of the TERT and regulate TA	[104]
ERG1	JAR and JEG-3 / HUVEC	Can act as an activator or repressor depending on the tissue of origin	[105,106]
ESTROGEN RECEPTORS	In sílico using Hex 8.0	Induced by the transcriptional activation of *hTERT* via *POT1* and *BCLX*	[107]
FGFR2	Glioblastoma	*hTERT* and *FGFR2* gene fusions activate tumor progression in malignant glioma	[108]
FOXO3	HGC-27 and U2OS	Contributes to *hTERT*-promoted = cancer invasion	[109]
GABPA and GABPB1	U-hth-74, U-hth-104, and MDA-T41	Activate mutant *hTERT* promoter and make telomerase activation	[110]
GDF11	Neuro-2a	Deletion of *GDF11* causes shortening TL, downregulation of TERT and TERC as well as reduction of TA	[111]
GLI	gastric cancer tissue	hTERT upregulated *Gli1* promote invasion and metastasis	[112]

GOLPH3	Pancreatic tissues	Promote cancer growth by regulating TA.	[113]
HIF-1 AND NANOG	Breast cancer stem cells	*HIF*-1 recruits *NANOG* as a coactivator for *hTERT* gene transcription	[114]
HKR3	Huh-7, Hep3B AND HepG2	Regulate cell cycle through *hTERT* inhibition and *CDKN2A* activation	[115]
HOXC5	NCI-60	Repress *hTERT* expression and tumorigenesis	[116]
HP1	ECR-293	Repress tumorigenicity	[117]
IL6	DLD1 and HT-29	Activate *STAT3*, *STAT1* and *NF-kB* by formed triplet complexes with *TNF-α* and increase TA by bind *hTERT* promoter site	[118]
KLF2	Primary human T cells from peripheral blood	Regulates *hTERT* expression: both to increase and to repress its expression	[119]
KLF4	VA13	Maintenance of telomerase activity	[120]
KMT2A	A375, MeWo, A431, WM35	Promotes cellular growth	[121]
MAD1	Bone marrow samples	*Mad1* reduce *hTERT* expression	[122]
MATRIX METALLOPROTEINASES	U2OS and VA-13	*hTERT* increased cell adhesion and migration	[123]
MCL-1	HEK 293T/17, U2OS, and HeLa	Mitochondrial pathway of apoptosis	[90]
MELATONIN	RS4-11 (MLL-AF4+ B-ALL) and Nalm-6 (non MLL-r B-ALL)	Suppresses cell growth and induced apoptosis, disrupt binding of *RBFOX3* to the *hTERT* promoter and *NF-κB* to the COX-2 promoter, suppressing *hTERT* and *COX-2* expression.	[124]
MENIN	U2OS	Downregulate *hTERT* expression	[85]
MZF2	U2OS	Downregulate *hTERT* expression	[85]
NEIL3	Bone marrow	Repair DNA damage in telomere regions *in vitro and* act as telomere-protecting protein.	[125]
NF-KB	U937 monocyte and murine RAW 264.7 macrophage and SK-N-MC	Regulatory mechanism between chronic inflammation and TA, cellular differentiation, tumorigenesis, and apoptosis	[126,127]
NFX1	HFKs	*NFX1-123* with *c-Myc* activate *hTERT* promoter activity, and NFX*1-91* can reduce *hTERT* expression	[128]
NME2	HT1080, HCT116, and MRC5	Epigenetic suppression of *hTERT*	[129]
NOVA1	H1299, H920, Calu6, HeLa, A549, HCT-116, H2887, H82, SHP-77, 293T and H2882	Take part of the splicing of *hTERT* and regulate they expression	[130]
NOXA AND PUMA	HepG2 and HL7702	They are regulated by *hTERT* promoter and can mitigate cell growth and promote apoptosis	[131]
NRF2	Mice lung tissues	*Nrf2* can negatively regulate ferroptosis via modulation of *hTERT* and *SLC7A11*	[132]
NUCLEOSTEMIN	HeLa	Cellular proliferation and maintenance of the telomerase complex	[133]
P53	MCF-7	Protect against DNA damage	[84]
P73	H1299, 293T, U2OS and BJ	Can act as repressors and activators of *hTERT* expression	[134]
PARP	MCF-7	Protect against DNA damage	[84]

PAX 5	Nalm6	Bind to regions proximal to *hTERT* translational and activate transcription	[135]
PAX 8	SF268, T98G, A172, LN-18, and U87MG	Regulation of TA by activate hTR promoter.	[136]
PGC-1B	Atm, Terc and TERT-ER mice	Low *hTERT* levels triggers vulnerabilities that difficult mitochondrial maintenance and increase oxidative defense mechanisms to promote anti-telomerase therapy	[137]
PINX1/NPM	HepG2, HEK293T, HeLa	*NPM* is recruited to *hTERT* by *PinX1* and is required in the proposed telomerase modulating unit to activate telomerase when telomere extension occurs during S phase	[138]
PITX1	293T, A2058 and GAK	Suppresses *hTERT* transcription	[139]
PPARγ	pulmonary arterial smooth muscle cells	*PPARγ* activation inhibits *PDGF* inducing proliferation and migration by modulating *hTERT*	[140]
PTBP1	H1299, H920, Calu6, HeLa, A549, HCT-116, H2887, H82, SHP-77, 293T and H2882	Axialites splicing of *hTERT* and regulates they expression	[130]
RB	U2OS	Downregulate *hTERT* expression	[85]
RBFOX3	MGC-803, MKN45, AGS, SGC-7901, and BGC-823) and GES-1	Promote cellular growth by activating *hTERT* signaling	[141]
RBFOX3	Hep3B, QGY7703, HepG2, SNU-44, N9 MG, U138, U251 and U373(59)	Promote tumor growth and progression via *hTERT* signaling	[142]
RFPL3	H1299, A549, H1975 and HBE	Acts as a specific transcription factor to control *hTERT* expression	[143]
RIF1	Ovarian cancer tissue	*RIF1* knockdown decrease the expression of *hTERT* reducing cell growth. Also act inhibiting promoter regions of hTERT.	[144]
RIN1	MCF7, MCF-12, MBA-MD-231	*RIN1* downregulating TA during *EGF* stimulation which results in inactivation of *hTERT* transcription.	[145]
SAHH	HUVECs	Inhibition of *SAHH* promote senescence via *hTERT* downregulation	[146]
SMAR1	Colorectal cancer	Downregulation of *SMAR1* activate *hTERT* increasing cancer stem cell phenotype.	[147]
SOD2	Atm, Terc and TERT-ER mice	Downregulation of *hTERT* reveals fragilities that promote anti-telomerase therapy	[137]
SP1	WI38, HFF and BREAST CANCER	Can act as repressors and activators of *TERT* expression	[148,149]
SP3	Fibroblast lines, 3C167b, 3C166a, GM639, GM847, and normal human fibroblasts	Actuate as promoter in *hTERT* resulting in the repression of this gene.	[150]
SPT6	CCD841, CCC-HIE2, RKO, LoVo, DLD1, SW620, SW480	*SND1* Control *hTERT* expression	[151]
STAT3	HepG2, MCF-7, DU-145, K562, A172, HS27	*STAT3* activates *hTERT* promoter together with *CD44* and *NFkB*.	[152,153]
STAT5	ILT-Hod, K562-ADM, K562	Activation of telomerase expression	[154]
SUMO E3	breast cancer cells	Regulation of *hTERT* expression and increasing migration and invasion	[94]
survivin	SW480, LS180, HT-29, Caco2, and Colo320HSR	Transcriptional Regulators of *hTERT*	[155]
TFE3	NCI-H1299	*TFE3* enhances cell cycle and cancer progression by binding to the *hTERT* promoter	[156]

TNF-A	Primary human ASM cells	*hTERT* expression decreases *TNF-α* expression	[157]
TNFAIP8L2	colorectal cancer	*TNFAIP8L2* inhibits TA by regulating *c-Myc* and *c-Est-2* binding to the *hTERT* promotor.	[158]
TNF-ALFA	DLD1 and HT-29	Activate *STAT3*, *STAT1* and *NF-kB* by formed triplet complexes with *TNF-α* and increasing TA by bind *hTERT* promoter site	[118]
TOE1	HeLa and 293T	Regulates telomerase maintenance	[159]
TP53	chronic lymphocytic leukemia patients	Evolution of *TP53* abnormalities is correlated with TL changes	[160]
TRIM28	UMG12 cells	*TRIM28-TRIM24* complex is recruited to the *hTERT* promoter mutation site through the *GABPA/B*1 complex and that the phosphorylation of *TRIM28* is required to activate *hTERT* transcription by releasing *TRIM24* from the mutation site	[161]
TRIP4	HeLa, SiHa, C33- A, DoTc2, HeLa S3, Caski, and Ect1	Promotes tumor growth and metastasis and regulates radio sensitivity	[162]
USF	Human normal peripheral blood mononuclear cell/SKBR3, MDA-MB-231, MCF7, 293T, SKOV3, Wi38, HFF AND BJ	They are related with regulation of *hTERT* expression	[163,164]
USF1/2	OEC-M1 and OC-2	Can act as repressors and activators of *TERT* expression	[165]
WNT	Xenopus laevis embryos	Transcriptional regulation, chromatin remodeler and proliferation	[93]
WT1	U2OS	Downregulate *hTERT* expression	[85]
ZEB1	MCF-7 and MDA-MB-231	Transcriptional activator of *hTERT* promoter sites and is involved in cell proliferation	[166]
